# “You’re just constantly on alert”: Women and Gender-Diverse People’s Experiences of Sexual Violence on Public Transport

**DOI:** 10.1177/08862605231186123

**Published:** 2023-07-19

**Authors:** Jessica Ison, Kirsty Forsdike, Nicola Henry, Leesa Hooker, Angela Taft

**Affiliations:** 1 La Trobe University, Bundoora, VIC, Australia; 2 RMIT University, Melbourne, VIC, Australia

**Keywords:** sexual harassment, sexual assault, sexual assault < GLBT, GLBT, stalking

## Abstract

Sexual violence is a public health issue that can be experienced across the life course. Public transport is a key site of sexual violence and harassment experienced by women and gender-diverse people in Australia, although victim-survivor voices have rarely been sought in addressing this issue. Through in-depth qualitative interviews with 41 diverse female and gender-diverse victim-survivors who were staff or students at two Australian universities, we sought to understand their experiences of sexual violence and harassment on public transport. We found that women and gender-diverse people, while often reporting on a significant experience of sexual violence or harassment on public transport, also had other, “everyday” experiences across their life course that impacted how they traveled and their confidence in the world. Overall, we argue that the significant impact of sexual violence and harassment on public transport should be addressed through targeting public transport as a key site for primary prevention of sexual violence and harassment.

On January 16, 2019, Aiia Maasarwe, a Palestinian-Israeli university student, was brutally raped and murdered by a 20-year-old man after she exited a tram. She was heading back to her on-campus residence in Melbourne, Australia. This was the latest in a string of murders of young women across Melbourne and a stark example of the threats women face every day in their journeys to and from home. Public transport is a known hotspot for sexual violence and harassment, particularly against women, girls, trans, non-binary, and gender-diverse transport users (Gekoski et al., 2017). While there is a growing body of research that employs a gendered lens to consider women’s safety on public transport in relation to sexual violence and harassment, there have been few qualitative studies focusing on the experiences of women and gender-diverse people on public transport (Ceccato et al., 2022). To respond to this gap in the literature, our study sought to explore university staff and student experiences of sexual violence and harassment on public transport. We conducted semi-structured interviews with women and gender-diverse participants from two universities in Melbourne, Australia.

The first section of the paper provides a brief overview of the key findings from the literature reporting on the prevalence of sexual violence and harassment, including on public transport. The second section summarizes the public transport context of this study and the method chosen to explore experiences. The third section reports on the key findings of the current study and the final section provides a discussion of the findings.

## Sexual Violence and Harassment on Public Transport

Globally, an estimated 6% of women have been subjected to non-partner sexual violence at least once since the age of 15 years (World Health Organization [WHO], 2021). However, these statistics may be higher for women facing other forms of structural inequality, such as racism or ableism (Townsend et al., 2022). Sexual violence is an umbrella term that the WHO defines as “any sexual act, attempt to obtain a sexual act, unwanted sexual comments or advances, or acts to traffic, or otherwise directed, against a person’s sexuality using coercion, by any person regardless of their relationship to the victim” (WHO, 2013). As a form of sexual violence, sexual harassment refers to

any unwelcome sexual advance, request for sexual favors or conduct of a sexual nature in relation to the person harassed in circumstances where a reasonable person would have anticipated the possibility that the person harassed would be offended, humiliated or intimidated. (Sex Discrimination Act, 1984)

In Australia and elsewhere, there has been a significant shift towards primary prevention of sexual violence and harassment which goes beyond responding to the issue to addressing the root causes of gender inequality (Our Watch, 2021; WHO, 2019). Public transport is a key site where primary prevention could occur (Hooker et al., 2020; Our Watch, 2021); however, there has been little uptake of primary prevention interventions by public transport providers to date (Ison & Matthewson, 2023).

Public transport is a known hotspot for sexual violence and harassment (Loukaitou-Sideris & Ceccato, 2020). Women and girls also face sexual violence and harassment on the station platforms (Chowdhury & van Wee, 2020), in vehicles, and during the last kilometer home. The last kilometer, or last mile, refers to the journey from the public transport stop to a person’s home or final destination, usually labeled a “whole of journey approach” when investigating safety and other issues of public transport (Natarajan et al., 2017). Women are more likely to be transport-dependent than men (Loukaitou-Sideris, 2016) and more likely to use public transport in more complex ways (Levy, 2019). This is called “trip chaining,” where women use multiple forms of transport to run errands, for care labor, and for commuting to work (Sánchez de Madariaga, 2012).

In terms of sexual violence prevalence, Whitzman et al. (2019) surveyed 517 tertiary students in Australia and found that almost 79.4% of female respondents had experienced sexual harassment on public transport, with similar rates for lesbian, gay, bisexual, transgender and queer (LGBTQ+) respondents. In comparison, 51.7% of male respondents reported victimization. Another Australian study of sexual assault and harassment at a university setting conducted in 2016 had over 30,000 respondents from 39 universities (Australian Human Rights Commission, 2017). When reporting on sexual assault or harassment experienced in a university setting, respondents could select the location where the sexual assault or harassment occurred, including the option “travelling to or from university.” Respondents reported that in the period of 2015 or 2016, transit was the most common location where sexual harassment occurred (22%). It was also a significant location of sexual assault (15%).

However, it is difficult to determine the true prevalence of sexual violence and harassment on public transport globally, or in Australia. As is the case with sexual violence broadly, victim-survivors may be reluctant to report their experiences due to mistrust of the police and authorities (Daly & Bouhours, 2010; Johnson, 2017). For example, in a U.S. study, 77% of 140 female tertiary students reported experiencing sexual harassment on public transport, yet only 4% reported to authorities (Natarajan et al., 2017).

Another difficulty with assessing prevalence is the large disparity between how studies measure prevalence. For example, in their rapid review, Gekoski et al. (2017) found that among the studies examining the prevalence of women reporting unwanted sexual behavior on public transport, there was a range between 15 and 95%. The discrepancies may be due to methodological limitations, including differing measurements of sexual violence and harassment, samples, methods, country- or city-specific factors, and discrepancies in what constitutes sexual harassment.

Even harder to quantify is the fear of violence, because sexual violence and harassment on a woman’s public transport journey also includes the threat or fear of violence. To address this, some studies have also focused on perceptions of safety on public transport (Carver & Veitch, 2020). For example, a U.K. study found that women’s fear after dark on public transport was 2 to 3 times that of men (DoT data cited in Loukaitou-Sideris, 2014, p. 245). Other studies echo these findings, including in Australia, where the Australian Bureau of Statistics’ *Personal Safety Survey* found that 87% of men felt safe waiting alone in the dark for public transport compared to 68% of women (Australian Bureau of Statistics, 2017). A Melbourne study surveyed 297 adults and found that 39% of women felt unsafe on public transport compared to 20% of men (Carver & Veitch, 2020). When looking specifically at night-time, 70% of women felt unsafe compared to 44% of men. And in a study of five different global cities, Plan International and Monash University (2018) found that 23% of young women believed it was unsafe after dark, particularly when traveling alone.

Alongside investigating prevalence and location, research has also looked at specific times when people feel more unsafe on public transport. In their systematic review of crime and safety in transit environments, Ceccato et al. (2022) found that while there are considerable safety concerns during peak hour, women in particular reported feeling most afraid in the evenings. Similarly, public transport users could have negative experiences when vehicles have a high density of users (e.g., Beller et al., 1980), although conversely, women also often experience fear when transport vehicles or platforms are empty (e.g., Vanier & D’Arbois, 2017). In addition to the fear they experience, women also spend considerable energy on strategies to keep themselves safe or change their own behavior, such as regularly changing their route (Lea et al., 2017; Stark & Meschik, 2018).

Women also face compounding issues, such as racism, ableism, homophobia, or transphobia. We note that there is little research on intersecting inequalities. Firstly, research on trans and non-binary people’s experiences is limited, but the emerging evidence shows these communities experience high rates of sexual violence on public transport (Gekoski et al., 2015). For example, Lubitow et al. (2017) conducted a qualitative study with trans and gender nonconforming people who use public transport in Portland, Oregon, a city in the United States. They found that trans and gender-nonconforming people had a heightened risk of sexualized violence. This was particularly heightened for people who experienced other forms of inequality, such as racism. A problem with the research on LGBTQ+ experiences concerns limited sample size. For example, Santoro et al. (2020) surveyed university student transit users (*n* = 2,138) across four cities (Huddinge, Mexico City, San José, and São Paulo). Of these, only 4% (Huddinge) to 23% (São Paulo) identified themselves as LGBTQ+. The study concludes that there are some similarities between LGBTQ+ people and cisgender heterosexual women who use public transport, yet they advise caution due to the small sample size. The LGBTQ+ community is underrepresented in this field, and we can draw limited conclusions from the research.

Secondly, research on experiences of women from diverse cultural and ethnic backgrounds is also limited. For example, alongside the experience of LGBTQ+ people, Santoro et al. (2020) also looked at the different travel patterns of people of color. They found that in Mexico City, participants who were women of color had higher rates of victimization. It is clear that while sexual violence is a gendered issue, it also intersects with other forms of inequality. Our study, while focused on experiences of sexual violence and harassment, approached this issue with an intersectional focus.

## Study Context

In Australia, public transport is the remit of each state and territory, although there are rail and bus services that do cross borders. In Victoria, the Department of Transport and Planning operates the state-based transport network through the front-facing Public Transport Victoria. Within the city of Melbourne, the network comprises trains operated by Metro Trains, trams operated by Yarra Trams, and buses operated by a variety of bus services (private and commercial), including a night bus service. Outside the metropolitan region, VLine trains service delivers transport in regional Victoria alongside different bus operators. The universities located in Melbourne are serviced by multiple modes of public transport, including bus, tram, and train, which expand across the Melbourne area.

There are a range of safety initiatives across the Melbourne transport network, such as emergency communication points, lighting, closed circuit television, and safety access points. In 2011, the Victorian Government implemented a new initiative to ensure that two officers are deployed after 6pm at every metropolitan train station and select regional stations. These officers, known as Protective Service Officers (PSOs), are part of Victoria Police. PSOs have similar powers to police, such as the power to search and arrest, and they carry firearms and capsicum spray. There are also Authorised Officers, otherwise known as ticket inspectors. They patrol vehicles and platforms or stops. They have the power to check for validated tickets and to issue fines.

## The Current Study

The current study aimed to understand women and gender-diverse student and staff experiences of sexual violence and harassment on public transport. We also wished to address the direct context of safety for university staff and students, given the rape and murder of Aiia Maasarwe. While there has been some research on sexual violence and harassment experienced by students on public transport in Melbourne (Whitzman et al., 2019), most studies use quantitative surveying, which mirrors much of the global research. This paper contributes findings from in-depth qualitative interviews to center victim-survivor voices. To that end, our primary research question was: “What are women and gender-diverse university workers’ and students’ experiences of sexual violence and harassment on public transport at Melbourne universities?”

## Method

### Design

We employed semi-structured interviews with women and gender-diverse workers and students at two different Melbourne universities. We adopted this method to draw on the diverse voices and experiences of victim-survivors (Brinkmann, 2020). The research design was reviewed and approved by the La Trobe University Human Research Ethics Committee (HEC19293).

### Participants

We undertook purposeful sampling and recruited from the two universities on the tram route Aiia Masaarwe took immediately before she was raped and murdered. We put up posters around the two campuses, sent emails to student and staff groups, and shared study details in lectures to inform potential participants about the study. Participants were also recruited through snowballing. Those interested in the study and wanting to share their experiences contacted the first author. The first author explained the study to those expressing interest and checked that the potential participant wanted to participate and was able and safe to do so, given the sensitive nature of the topic being explored. Participants were eligible if they were female, non-binary, or gender-diverse, were currently enrolled and/or working at one of the two universities and had an unwanted sexual experience on public transport. However, the experiences we asked about could have occurred at any time on public transport, not just during their journeys to or from campus.

In total, 41 people participated in semi-structured interviews for this study. This included: 6 staff members and 31 students, as well as 4 participants who identified as both a staff member and a student. Of the student participants, three lived on campus, seven were international students, and one was Aboriginal and/or Torres Strait Islander. In relation to gender, 39 participants identified as female, 1 as non-binary, and 1 as “no gender.” Participants were given an open text box to indicate their ethnicity, with the largest category (*n* = 7) identifying as Australian. Two participants did not specify an ethnicity, and the remaining 32 participants identified 19 different ethnicities. The age range was 18 to 50 years, with the majority (68%) of participants under 30 years. Regarding sexuality, 28 participants were heterosexual, 12 were lesbian, bisexual, pansexual, and/or queer, and 1 preferred not to say. Finally, 10 participants identified as having a disability.

### Procedure

Interviews of up to an hour were conducted in person at the university or via video conferencing and audio-recorded with consent (Interview protocol, Supplemental Appendix 1). Participants were provided with contacts for support services and a distress protocol was followed. All research team members attended training in responding to disclosures of sexual violence. Participants were given a $20AUD voucher for their participation in recognition of their expertise and time.

### Data Coding and Analysis

The audio recordings of interviews were transcribed and imported into the NVivo 12 qualitative software program (QSR International, 2013). We used reflexive thematic analysis to identify, analyze, and report on key themes across the dataset, drawing on our own subjective experiences and perspectives (Braun et al., 2016, 2019). This form of analysis was chosen to acknowledge our positioning and the reasons behind undertaking this study. A reflexive approach, as explained by Braun et al. (2016, 2019), also aligned with our own understandings of qualitative methodology as a means to explore stories and construct meaning, rather than to search for truth embedded in the data. As such we have chosen not to undertake either a “codebook” or “coding reliability” approach to our thematic analysis (Braun & Clarke, 2019, p. 845; Braun et al., 2019, p. 593).

We undertook a deep engagement with the transcripts, reading them several times and regularly discussing them in the process of constructing codes. We then developed codes across the dataset. From these codes we looked for patterns across the data which we built into themes. We continually reflected on these themes and their legitimacy throughout coding. We then drew out two themes that answered our research question. Firstly, “Experiencing sexual violence on public transport,” with the sub-themes “Impact across their lives” and “Perceptions of reporting.” Secondly, “Making sense of the impact” with the sub-themes “The emotional impact” and “The behavioral impact.”

In the presentation of quotations, we have replaced participants’ names with pseudonyms. Where participants speak of a specific area, details have been generalized to minimize the possibility of identification.

## Findings

Participants’ experiences of sexual violence on public transport varied widely, including stalking, sexual harassment including sexualized verbal assaults, sexual assault, physical violence, homophobic and transphobic verbal assaults, and racist verbal assaults. Participants reported a broad range of experiences not only on the mode of transport (tram, bus, train, and taxi/rideshare) but also on platforms, in transit, and during the last kilometer home. All participants reported that the perpetrator or perpetrators were male and unknown to the victim.

Participants’ perceptions on the safety of using public transport varied widely. Some felt more nervous at night, others during peak hour. Some participants felt that buses were safer than trams, while others preferred different modes of transport. However, all participants had an experience of sexual violence and harassment at some point during their public transport use which impacted how they took public transport and, further, how they felt in public in general. The following themes and subthemes were identified. Firstly, “Experiencing sexual violence on public transport,” with the sub-themes “Impact across their lives,” and “Perceptions of reporting.” Secondly, “Making sense of the impact,” with the sub-themes “emotional impact” and “behavioral impact.”

### “I don’t know where to start, there have been so many times”: Experiencing Sexual Violence on Public Transport

Participants were asked to describe the events they had experienced with the prompt “Is there a time when you personally felt afraid on public transport?” In response, participants would frequently say, “Yeah, quite often” (Kirra) or “Yes, multiple [times]” (Giorgia) or “I don’t know where to start, there has been so many times” (Maya). While participants were mostly motivated to participate in the study to share one specific incident, across the interviews they tended to refer to multiple incidents. A distinct trend was for the participant to then minimize times when it was not “serious” compared to the specific experience they had come to talk about. The experiences participants deemed less serious were often related to sexual harassment (sexualized language, touching areas other than their genitals, flashing genitals), while participants viewed sexual assault (touching of genitals, perpetrator’s genitals touching them) as more serious. With probing from the interviewer, some of these examples were talked about in more detail. Therefore, while they may have opted into the interview because they wished to talk about their experience of stalking or sexual assault, they also discussed numerous examples of sexual harassment during the interview.

When describing “less serious” experiences they would often talk in general terms rather than provide specific details, such as Maya, who said:

so usually it’s . . . especially at night, there’ll be a billion seats and then some guy will sit right next to me, and they always just make a point somehow to make some body contact with you. If I’ll move a little bit even closer to the window, they’ll move . . . yeah, gross . . . Then obviously, when I was younger, when people used to talk to me, I just felt I’ve got to be polite and talk back, because I was scared that maybe they would get a bit more angry and stuff. But now I’m just in full ignore mode. I don’t care. Get out of my life. I’m not fucking dealing with this.

Using a generalized description of sexual violence and harassment highlights not only how normalized sexual harassment is on public transport but also how participants themselves have collapsed their experiences together.

Maya’s experience is similar to other participants: typically, a man comes up to them, sits close, and starts talking. Others gave examples, such as, “You’re a beautiful girl . . . you should come work with me in [location]” (Bianca). This is often followed by asking for their details, “Can I have your number, I’ll text you” (Bella). Most of the participants also described how they attempt to deflect the approach or encounter by being polite and non-confrontational:

as a female I kind of feel like you just have to be polite back, so you don’t provoke anything, and if you don’t be polite I feel like it’s instilled into us, ingrained into us, you have to be nice to men otherwise you’re going to provoke something or you’re going to escalate it. (Kelly)

The focus on not provoking men was paired with the difficulty of removing themselves from the situation, often because they may be pinned in their seat or against the wall of the carriage, which meant they had to find an excuse to try and leave, or engage in another activity, such as reading a book or looking at their phone, in the hope that the person would stop talking to them.

Alongside sexual harassment, participants wanted to talk about a specific incident, and these tended to be stalking or sexual assault. In terms of stalking experiences, multiple participants described being approached by a man who cornered them in a seat. They described moving to another part of the vehicle and being followed by the man. For some participants, this happened multiple times, and as they came close to their stop, they adopted a range of strategies, such as calling a friend or family member to meet them at their stop or getting off at a well-lit stop or at a shop where they could hide. In one instance, Saskia approached some Authorised Officers who boarded the tram and asked them for their assistance. They shielded her so she could surreptitiously leave the tram. A report was made to the police who subsequently looked at security footage and found that the man had stalked Saskia throughout the area she was walking for at least fifteen minutes and then onto the vehicle.

Stalking can also happen beyond the initial incident. For example, Ryley was approached at a transport stop by a man. He followed her onto the vehicle, trying to talk to her. She was afraid and eventually exited at a place where she felt safe. She tried not to take public transport for a few days. However, over the following weeks, the stalking escalated. She tried to report to the police, yet they dismissed her. A short while later, he appeared at her place of work. Again, the police were dismissive. In the first instance, she thinks she saw him take photographs of her. She believes he used technology to stalk her beyond the public transport stop.

Participants also disclosed experiences of sexual assault. This included non-consensual touching on parts of their body, including their genitals, or a man rubbing his genitals on their bodies. For example, one participant had a driver touch her genitals as she maneuvered her wheelchair onto the vehicle (Zoe) and others were sexually assaulted by other public transport users. Often this was relayed in very matter-of-fact descriptions such as:

I was on a tram alone, it was on a weekend, . . . and I was sexually assaulted on the tram on the way to—I was heading towards [location], and the police were called and there was a police report and all that jazz, and the—it was a man and he was very mentally unwell it turned out. (Jing)

While participants all had experiences of sexual violence or harassment on public transport, this was often compounded by other issues. For example, women who are transport-dependent and who have children, particularly in prams, face the issue of regularly needing assistance from strangers. As such, women may organize their route to avoid public transport. Amina indicated this:

For me tram is not convenient [and neither is the bus], I got yelled once by bus driver because I took so long when I wanted to get off. I went with the pram and the bus driver didn’t lower down . . . the bus. Sometimes I still feel traumatized if I want to [seek assistance].

Travelling with a pram can mean that participants were forced into situations that were outside of their control.

Another compounding issue participants reported was being targeted racially and/or the basis of their religion. For example, three participants were Muslim women and reported that they had experienced vilification based on their religion and/or ethnicity, which had made them feel unsafe. As explained by Laila, “I feel like being Muslim, and also female, is twice as hard.” Laila spoke further about this sexualized abuse that was also based on racist stereotypes or racist slurs, saying:

a couple [men] . . . came onto the bus and sat at the back, like right next to me, and they had a massive boombox type of thing with them, and they were all playing, like, racist rap, like things that were specifically very misogynistic and racist, and I felt like I—like, even when I moved closer to the bus driver, to the front of the bus, I felt like they were looking at me, and it just was this icky feeling.

This indicates that women of color are at a heightened risk of being targeted because of their race and/or religion as well as their gender. As Yan commented, it was a “weird combination” of racial and sexist abuse.

Others felt targeted because of their presentation of gender or sexuality. For example, Dex talked about how they are targeted differently as either “a dyke or a faggot,” depending on how they are dressed or how they presented themselves, and that when someone is confused about their gender or sexuality, Dex said it often turns into “quick anger.” This experience was echoed by Lara, who talked about the sexualized attention she has experienced when she was with a partner in what people assumed to be a lesbian relationship: “we’d get a lot of attention on public transport that was quite sexual because we were together, like in terms of stares and sort of just body language and smiles and stuff like that.”

Alongside racist and LGBTQ+ specific experiences, participants with a disability also experienced specific forms of abuse and harassment. Zoe stated that, “I drive because often I cannot get on the bus with my wheelchair. Mainly, I’ve had to wait up to three buses to get on a bus because they don’t know how to [get the wheelchair ramp out]” and waiting could be dangerous and scary. Zoe also talked about the specific experience women with disabilities may have where staff may touch them without consent to assist them. She explained:

I’ve had so many bus drivers, thinking it’s okay to touch me, not in a sexual manner . . . but males put their hands on my shoulders and trying to push me when clearly [my wheelchair] is electric. I’m often touched and no it is not sexual, but I do wonder why they do it . . . It feels creepy . . . it’s my body and it doesn’t work like yours but it’s still my body and I’m still human.

A final element of the experience of participants was the disappointment many felt when no one intervened. For example, Maya said, “There were so many people around and no one got involved. That’s what I noticed. It was the weirdest part.” Participants were left to grapple not only with the events but also their disappointment and sadness over the lack of help from bystanders.

However, there were examples of participants who intervened on behalf of someone else, and then became a target. For example, Courtney intervened when a man was racially abusing another passenger. The man then turned to abuse her, and no one assisted her. She said:

I don’t regret that I did it [intervened] but I really regretted the aftermath because then he just became focused on me instead and that was a really busy bus because it was 4.30 in the afternoon, but nobody wants to get involved.

As mentioned above, across the interviews, while participants intended to discuss one incident, many arose that highlighted the everyday nature of sexual violence and harassment. Such experiences also impacted, and were impacted by, their race, religion, or other intersecting aspects of their lives.

### “Kind of skews my experience of the world”: Impacts Across Their Lives

Most of the participants in our study also spoke about other experiences they had had, which were not public transport-specific, but impacted their experiences of unwanted behaviors on public transport. For example, Frankie explained that “having experiences during my childhood and adulthood of being sexually assaulted and sexually abused, like that kind of skews my experience of the world.”

Many participants also talked about the age they recall sexual harassment happening when they were younger. For instance, for Olivia, “oh probably 10, I’d say” or Giorgia “13, 14, that young.” Similarly, Alice recalled:

I started to notice it when I was about fourteen, fifteen and I’d be catching public transport to go out for dinners with my friends or I’d go to the movies, and you know, the amount of marriage proposals that I got when I was like in Year 9 and Year 10 was just unbelievable.

A consistent trend was participants recalling sexual harassment or assault happening while they were in high school:

I feel like it was almost . . . you were almost even more of a target if you were in school uniform compared to being in normal plain clothes. You’d just be even stared at. Every morning if a car was driving past you, you’d just be stared at or yelled stuff at. It’s disgusting. (Kelly)

Alongside their own experiences, most participants also shared incidents that happened to their friends. For instance, Kirra said, “[It happens] all the time. My best friend constantly. She got asked for a threesome the other day when she was going home from work.” Olivia commented that, “a lot of my friends had the same stories” and Kelly mentioned, “So it actually happened to my friend a few days ago” (Kelly). Also, Alice said, “a girl that I went to high school with . . . she was actually raped at [a busy train station] during the day.”

Overall, it was clear that participants’ lifetime experiences of sexual harassment and assault, both on and off public transport, paired with the experiences of friends and acquaintances, impacted how they took public transport. As Olivia said, “having all those experiences, you’re just waiting for the next one.”

### “I don’t think they would take it seriously”: Perceptions of Reporting

Of the 41 participants in our study, only five had reported them, or had someone else report, to the police. In some instances, other transport users or public transport employees called the police. Those who did report to the police, did not have positive experiences. For example, Ryley said, “I was pretty unimpressed with their response. They were very dismissive, they pretty much as soon as I’d said, ‘no he hadn’t actually touched me,’ they just really couldn’t care less.” None of the participants we spoke to sought the assistance of PSOs.

When asked whether they would go to the police, participants simply responded, “No, I don’t think I would. I don’t think they would take it seriously, I’m pretty sure” (Olivia). The reasons for not reporting were varied, but a general concern was that the incident was not worth reporting: “it’s just not something that I would think to bother the police with” (Courtney). Most participants felt they would not report to the police or PSOs, unless it was what they deemed serious: “If something physically happened and I was genuinely worried for my safety, yes. But if it was harassment, no” (Kirra). This was due to the belief that only provable physical violence would be taken seriously:

You just feel like unless you are assaulted, nothing is going to happen. Anything that isn’t physical and even then, unless you can really seriously prove it could only have been this one person, nothing is going to happen. (Giorgia)

For some, the decision not to report was directly shaped by previous negative experiences of reporting to the police. Others had negative experiences themselves with the police, such as Dex, “I have been seriously assaulted by cops. And then I had to go to court. So fundamentally [I] don’t trust them because they assaulted me quite badly when I was arrested.” Or one participant reported having “lewd looks even from policemen” (Tara). This history with the police extended to fears of PSOs:

it terrifies me that they carry guns. And I don’t see them as a safety network. I see them as a group of people who aim to make profit, and meet quotas of people who haven’t bought their tickets. I don’t see them as safety officers. I see them as ticket checkers who fine you. (Dex)

The perception that PSOs are unsafe was echoed by other participants: “They’re not there to make anybody feel safe. And they’re basically, going in with like, this pack mentality on lone passengers, they are fucking arseholes” (Lauren). Another participant said she did not trust the police and when asked if this extended to PSOs she said: “They’re worse, I hate them” (Lara). On the other hand, some participants held positive perceptions about the PSOs:

I do feel safer that they’re there though. So, I’m glad they’re there. I just wish they were trained in mental health or trained in some kind of intervention. They just seem like, they’re like posts to being intimidating and that’s it. (Kirra)

Participants also questioned the role of PSOs, with them stationed only on platforms:

PSOs I just see as standing on the platform, they don’t actually really make me feel any safer, because I don’t feel like if someone’s going to target me it’s going to be on the platform. (Lara)

A few participants said they felt safer with PSOs at the platforms in the evenings, like Giorgia who said, “I really like the fact that they’re now at every station after six o’clock.”

Alongside the police and PSOs, there are also alternative reporting options provided by transport organizations. However, only one interviewee reported to a public transport service. The reason she reported is because the perpetrator was a public transport employee who had sexually assaulted her, but the employee retained his employment because the police said there was insufficient evidence to take the case further.

### “Public transport is my only option”: Making Sense of the Impact

Participants’ negative experiences on public transport often impacts them in other aspects of their life. Some participants had been so terrified by the incident or incidents that they were severely restricted and afraid to leave the house. Those who were still public transport-dependent reported planning their day around feeling safe when traveling. This planning involved such strategies as picking the safest route, considering when they could run errands, whether they could attend an event after dark, and what they should wear to avoid attracting attention. Some participants chose to avoid all public transport, making monetary sacrifices to maintain a car or taking taxis. However, this was not a possibility for some and therefore they had no option but to organize their life around safe transport options: “Public transport is my only option, and it’s not safe so what should I do, should I just fly?” (Amina)

The impact of planning one’s life around public transport is particularly evident for female students who choose a class or a subject depending on the time the subject is available, rather than their preferred choice: “I just think people don’t realize, like, the sheer planning that goes into—I have to plan my whole career and my whole studies and everything else around personal safety” (Rachel).

As noted above, the story participants came to share with the interviewer was generally one of many experiences across their life. As such, it was not just their experience(s) of sexual violence and harassment on public transport that impacted them. As Alice said, “there’s just been a lot of experiences that I’ve had that have sort of lead me to sort of avoiding [taking public transport].” To look further at the impacts of sexual violence and harassment, we separated it into emotional impact and behavioral impact. However, these overlapped considerably.

### “I mean to be honest, you just learn to ignore it”: The Emotional Impact

Overwhelmingly, participants felt fear from their negative experiences, both in the short and long term. This fear made them alter their travel but also their life: “Then after [being stalked on public transport] basically I didn’t go back to work, and I didn’t get on a tram again for a couple of years actually” (Ryley). If participants were too scared to speak out, they also felt guilty and as though they were to blame:

[Standing up to injustices] are my values, but I’m not living my values because I’m scared that if I do, my safety will be impacted if I speak up for myself. Then I guess, what you permit you promote. (Maya)

Participants were asked about whether they had heard of any of the media stories of attacks against women in Melbourne and if so, how those stories had affected them. Muslim students were particularly impacted by the murder of Aiia Maasarwe: “When I went to the mosque, most of the sisters, they were talking about Aiia’s incident, and how afraid and scared they were at that time” (Djamila).

Most of the participants also mentioned other high-profile cases in Melbourne. All of the participants were impacted in some way, from being more cautious through to changing their route and avoiding the places where the women were murdered. Many felt there was no difference between themselves and women who have been murdered. Participants mused that it could be them next:

There’s no reasonable difference between her and me, like it is just time and place, and . . . it really is like you just have to put it on the list of other kind of probabilities or random ways that you could be injured or die, is like a man will come and kill you. (Savannah)

Participants were also often angry that sexual harassment and assault is the norm for women:

I get angry that we have do that and that there’s a real unawareness of that, you know, by men. And just the cultural norm I suppose, in that, that’s what it takes to be a woman and that’s that, you know, there’s no, . . . intolerance of that. (Jamie)

While angry, most participants also feel resigned to public transport being unsafe and to having to remain vigilant on every journey: “I mean to be honest you just learn to ignore it after some time. It doesn’t even feel weird anymore” (Areeba).

While being resigned to the issues, participants also voiced resilience and strength:

I have made a conscious decision to not let those kinds of reports or experiences prevent me from doing that. You do change your behavior to try and be safer while still being able to be out in the world. (Courtney)

### “You’re already really vigilant”: The Behavioral Impact

The impact on all the participants was also experienced in how they acted, how they dressed, what their movements were, and more. For example, Kirra said, “I tend to make sure I’m covered up or I don’t look like, look inviting, I guess. And I don’t smile on public transport anymore so that I can just be left alone.” Dex said they will often make a conscious decision around what to wear, and that “in summer this [is a] negotiation with staying cool but not showing too much skin.” Alongside their clothing, it can also extend to their hair. Diwa explained that:

My hair wasn’t always this short, it was actually really, really long, and what made me sort of go fucking nuts with my hair, and cut it, was because I was so fucking tired of getting sort of looked over by those middle-aged dudes on the train.

The impact also extended to how they maneuvered once they boarded the vehicle. In particular, it could create a feeling of hypervigilance. For example, Kelly said:

when you already have a situation like that, that’s happened, you’re already really vigilant, and you’re looking at everyone around and you’re like is he or she going to come up to me, what are they going to say. You’re just constantly on alert.

This state of being alertness means participants assess all aspects of the trip, which Sarah described as: “Where am I? Where’s the door? Who’s around me? Who can I trust? How can I? What if? What will I? All of that kind of stuff.”

The perceived necessity of hypervigilance also meant that they could not relax on their public transport journeys:

It’s definitely taken quite a bit of my brain space, I guess. Like, when I’m on public transport, I’m always sort of on alert, kind of thing. Like, I never let myself take a nap or anything, especially on really long public transport. (Diwa)

This can also impact their feelings of self-worth:

I was just thinking back to why I haven’t really taken public transport that often, and there are these things that you internalize, and you don’t quite unpack it, and it’s only until you recall all these isolated things that you realize that it’s all because of these few reasons, that has unconsciously influenced the way that I make choices. (Laila)

## Discussion

Women and gender-diverse people commonly experience sexual violence on public transport. Participants in our study described events that have left a lasting impact on them, such as stalking and sexual assault, as well as “everyday” experiences of sexual harassment. They also discussed their past experiences of sexual violence and abuse across their lives, how this affected their use of public transport, and their willingness to report their experiences.

All the participants said that the perpetrator was an unknown male (or unknown men). The evidence on sexual assault outside of public transport indicates the perpetrators as most likely known to the victim (Australian Institute of Health and Welfare, 2020). However, it is unsurprising that the forty-one participants we interviewed did not know the perpetrator as the study focused on unwanted public transport experiences. While sexual violence and harassment from known perpetrators undoubtedly occurs on public transport, popular understandings of the risk of sexual assault on public transport relates to an unknown male. Public transport is also one setting where an unknown perpetrator has the capacity to target a female stranger.

In general, there is considerable underreporting broadly of sexual violence and harassment to the police (Thompson et al., 2010). This is due to distrust of the police, previous negative experiences with police or other authorities, as well as fears of not being believed (Johnson, 2017; Jones et al., 2009). Issues with reporting extend to public transport. In their survey of 517 Melbourne university students, Whitzman et al. (2020a, 2020b) found that only 5.7% reported the incident to police or a transit authority. In another publication with the same data, Whitzman et al. (2020a, 2020b) compared victim reporting in Melbourne to three other major cities. Melbourne’s rates are similar to Rio Claro in Brazil (4%) and Milan in Italy (3%) but Tokyo/Kanagawa in Japan has a much higher rate of reporting (17%). The most common reason for not reporting by Melbourne service users was because “they did not think the crime was serious” (p. 246). They also note that respondents said “they did not think authorities would catch criminals” as a reason 40% did not report (p. 248). In our study, participants voiced a similar belief that their experiences were not serious enough and that police would not take it seriously. Some also indicated distrust of the police that extended to their own lived experiences of marginalization and previous negative police interactions.

The distrust of the police also extended for some to the PSOs, though other participants felt safer with the presence of PSOs. The distrust of PSOs is reflected in research conducted into their effectiveness. A report from the Victorian Auditor-General looked at the effectiveness of PSOs and concluded that there was evidence that the presence of PSOs created a higher perception of safety; however, “it is not possible, on the available data, to determine if their presence has had an impact on crime” (2016, p. vii). Alongside their lack of effectiveness, there has also been considerable reports of their misconduct. Notably, there was a spate of reports on PSOs using excessive force against transport users. This was investigated by the Victorian Ombudsman (2015). In relation to sexual violence and harassment, the investigation was also concerned that there had been reports of PSOs acting in predatory ways toward transport users, particularly young women. The Independent Broad-based Anti-Corruption Commission investigated this and found that the most common occurrence was “a PSO targeting a young woman to obtain personal details so they could then contact her socially” (2016, p. 17).

Studies have captured unwanted sexual experiences on public transport, but the impact has been little explored. Participants reflected on a range of ways that single incidents, as well as the experiences over their lifetime, impact how they engage in public transport use. Such stark examples of monitoring their own behavior as well as hyper-vigilance indicate an urgent need to prioritize greatly improved safety on public transport.

## Policy Implications

This paper highlights the complex interaction between sexual violence experienced on public transport and across the life course. There is no simple way to address this issue. Approaches need to range from improving training for transport staff to challenging misogynistic advertising present at public transport locations (TramLab, 2021a, 2021b). In Australia, there has been a shift towards addressing gender-based violence through primary prevention. From our research, it is clear that women and gender-diverse people experience multiple forms of sexual violence and harassment and that there needs to be a shift toward preventing perpetration. To achieve this, public transport needs to be a key site of the primary prevention approach (Ison & Matthewson, 2023). We recommend that public transport becomes a key site for intervention, and that an intersectional lens is required to address sexual violence and harassment (TramLab, 2021a, 2021b).

## Limitations

This study was limited to one city in Australia. It did not capture the experiences of women and gender-diverse people who live in rural areas. It was also limited to people who studied or worked at a university, though the sample included people with different levels of higher education, from first year students to those with postgraduate degrees.

Finally, the interviews were only conducted in English, limiting the participation of students with low English literacy. More research is needed on the experiences of sexual violence among structurally oppressed groups, including those with disabilities, international students, migrants, and Indigenous Australians.

## Conclusion

Women and gender-diverse people who use public transport, and particularly those who are transport dependent, have little choice but to continue taking routes where they may have had an experience of sexual violence and harassment. However, they also show great strength and resilience in the face of what for some feels like ever-present risk and fear. Public transport providers and governments need to urgently address this issue and invest in more research to understand and intervene effectively to prevent sexual violence and harassment on public transport.

## Supplemental Material

sj-docx-1-jiv-10.1177_08862605231186123 – Supplemental material for “You’re just constantly on alert”: Women and Gender-Diverse People’s Experiences of Sexual Violence on Public Transport Click here for additional data file. Supplemental material, sj-docx-1-jiv-10.1177_08862605231186123 for “You’re just constantly on alert”: Women and Gender-Diverse People’s Experiences of Sexual Violence on Public Transport by Jessica Ison, Kirsty Forsdike, Nicola Henry, Leesa Hooker and Angela Taft in Journal of Interpersonal Violence
