# Supplementary material for: “You’re just constantly on alert”: Women and Gender-Diverse People’s Experiences of Sexual Violence on Public Transport
Source: J Interpers Violence. 2023 Jul 19;38(21-22):11617–41. doi: 10.1177/08862605231186123 (PMC10515455; doi:10.1177/08862605231186123)
Supplement: sj-docx-1-jiv-10.1177_08862605231186123 – Supplemental material for “You’re just constantly on alert”: Women and Gender-Diverse People’s Experiences of Sexual Violence on Public Transport [file sj-docx-1-jiv-10.1177_08862605231186123.docx]

**Supplementary - Interview Guide**

1. Tell me about your use of public transport to and from this campus

[Prompt: why do you use it? walk me through your usual journey to and from the campus; how do you feel about this journey?]

**[Signposting guide for Interviewer: Now I am going to ask some questions about feeling unsafe while travelling, are you OK with me asking you about this, and are you happy to proceed?]**

1. Has there been a time where you have felt personally afraid?

[Prompt: In what way? Gently probe about any actions they may have taken.]

[Note: specific locations and time of the day and what days of the week]

**[Signposting guide for Interviewer: Now I am going to ask some questions about experiencing or witnessing unwanted sexual attention feeling unsafe while travelling. Are you OK with me asking you about this, and are you happy to proceed?]**

1. Have you ever felt personally afraid for your safety and/or experienced an unwelcome sexual advance or other unwelcome conduct of a sexual nature?

[Prompt: can you tell me a little bit about that? Was it someone you knew? Did anyone intervene? Did you report it? If so why? If not, why not?] If you did, how was it received and were you satisfied with the response?

1. Have you witnessed or heard about other women receiving unwelcome sexual advances or other unwelcome conduct of a sexual nature? Perhaps tell me a little bit about this.

[Prompt: Did they seek any response or support from anyone and what happened?]

1. How has this/have these experiences affected you?
2. Issues around women’s safety have been in the news a lot more recently, how has that affected you?

[Prompt: have you heard of Aiia Masaarwe? How did you react to this news in terms of getting to and from the campus? Or using public transport generally?]

1. What do you do to keep yourself safe using public transport?
2. How have your experiences of doing this journey differed from your other use of public transport?
3. What could anyone or any agency do to help you feel safer on the journey to and from campus?

**[Interviewer to provide list of resources for participant either at any point during the interview, and/or at the close of the interview, as required]**
